# Ubiquitin-specific proteases in inflammatory bowel disease-related signalling pathway regulation

**DOI:** 10.1038/s41419-022-04566-6

**Published:** 2022-02-10

**Authors:** Rirong Chen, Xiaobai Pang, Li Li, Zhirong Zeng, Minhu Chen, Shenghong Zhang

**Affiliations:** 1 Department of Gastroenterology, The First Affiliated Hospital, Sun Yat-sen University, Guangzhou, China; 2 Zhongshan School of Medicine, Sun Yat-Sen University, Guangzhou, China

**Keywords:** Inflammatory bowel disease, Ubiquitylation

## Abstract

The exact pathogenesis of inflammatory bowel disease (IBD), a chronic gastrointestinal inflammatory disease comprising Crohn’s disease and ulcerative colitis, remains unclear. Studies on ubiquitination, which regulates the degradation of inflammation signalling pathway molecules, and deubiquitination have provided novel insights. Targeting the ubiquitin-specific protease (USP) family of deubiquitinases elucidates IBD signalling pathway mechanisms and possibly, IBD therapeutic solutions. Here, we characterised USPs as chief regulators of pro-inflammatory signalling pathways, including nuclear factor-κB and transforming growth factor-β; analysed the relationship between USPs and IBD pathogenesis in terms of genetic susceptibility, intestinal epithelial barrier, immunity, and gut microbiota; and discussed future research prospects.

## Facts

- Inflammatory bowel disease (IBD) is a chronic intestinal disorder. Various signalling pathways have a regulatory effect on IBD, but the specific activation and regulation mechanism of the signalling pathway is still unclear.
- Deubiquitination implicates numerous biological processes and plays an essential role in various human disease generation.
- Ubiquitin specific proteases (USPs) are largest family of deubiquitinating enzymes and involved in the signalling pathway regulation as well as pathogenesis of IBD.
- The findings regarding the role of USPs in IBD may point out future researches and approaches to the therapeutic targets of IBD.

## Open questions

- What are the constituents and functions of USPs family?
- How USPs regulate the inflammation signalling pathways?
- What is the role of USPs in the pathogenesis of IBD?

Inflammatory bowel disease (IBD), a chronic gastrointestinal inflammatory disease comprising Crohn’s disease (CD) and ulcerative colitis (UC), has become prevalent worldwide [1]. Rising case incidence and pathological therapy challenges have recently led to extensive research on the pathology and treatment of IBD, a disease with precipitating factors such as impaired intestinal barrier, altered gut bacteria, and disordered immune responses [2]. Understanding the impact of these pathological alterations is key to discovering treatment targets, and multiple studies have focused on the signalling pathways involved in IBD pathogenesis to clarify the underlying mechanisms [3].

Despite recent studies on IBD mechanisms and pathways, a precise identification of signalling pathway targets has yet to be achieved. Previous researches have elucidated the function of enzymes in ubiquitination, a post-translational process that provides an important opportunity for the regulation of immune-inflammatory response signalling molecules [4]. However, deubiquitination, the process that reverses ubiquitination, has not been examined critically with respect to IBD regulation. Deubiquitinating enzymes (DUBs), a superfamily of proteases that can be categorized by catalytic mechanism into cysteine proteases (USPs, UCHs, OTUs, MJDs, MINDYs and ZUP1) and metalloproteases (JAMMs), are critical in regulating ubiquitin signalling [5, 6] and associated with human diseases such as cancer, centre nervous system disease, autoimmune disease and infections [7]. DUBs remove ubiquitin from specific substrate proteins and affect subsequent proteasomal degradation and sub-cellular localization, which maintains a balanced level of protein quality control and homoeostasis between ubiquitination and deubiquitination in physiological condition [7]. Ubiquitin-specific proteases (USPs) are the largest family of DUBs and play vital roles in deubiquitinating modification. The present study characterises USPs as the primary regulators of pro-inflammatory signalling pathways, including the nuclear factor-κB (NF-κB) and the transforming growth factor-beta (TGF-β) pathways. We analysed the relationship between USPs and IBD pathogenesis in terms of genetic susceptibility, immunity, intestinal epithelial barrier function, and gut microbiota and discussed prospects for future research on USPs and IBD.

## USP role and mechanism

### USP structure and catalytic mechanism

As the largest family of DUBs, USPs bear both similarities with, and critical differences from, the other cysteine protease DUBs. All DUB catalytic domains contain a primary ubiquitin-binding site that enables numerous interactions with ubiquitin in the distal region of a polyubiquitin chain [6]. USP catalytic domains, similar to those of classical cysteine proteases, rely on a catalytic dyad or triad of conserved amino acid residues; thus, the various isopeptide-bond hydrolysation processes of different USPs are quite similar to one another. In contrast to other cysteine protease DUBs, USPs contain specific USP domains exhibiting a functional catalytic mechanism and consisting of three conserved subdomains resembling the thumb, palm, and fingers of a right hand. The USP catalytic centre, which includes a C-terminal His Box and N-terminal Cys Box with catalytic His and Cys residues, respectively, is located at the interface between the thumb and palm subdomains, and the finger subdomain grips the distal ubiquitin in the ubiquitin chain during interactions [6]. USP family members have varying molecular structures, with most comprising a core catalytic domain with insertions and other N- and C-terminal extensions, which may consist of different domains or sequences and perform specific functions [8]. As examples, the USP5 N-terminus contains a ubiquitin-binding zinc-finger domain required for binding the ubiquitin C-terminus diglycine motif [9], while the USP7 N-terminal tumour necrosis factor receptor (TNFR)-associated factor domain recognizes various substrates, enhancing USP7 catalytic activity [10]. CYLD is the only USP family member that lacks the finger subdomain [11].

### Ubiquitin code and USPs

**Fig. 1:**
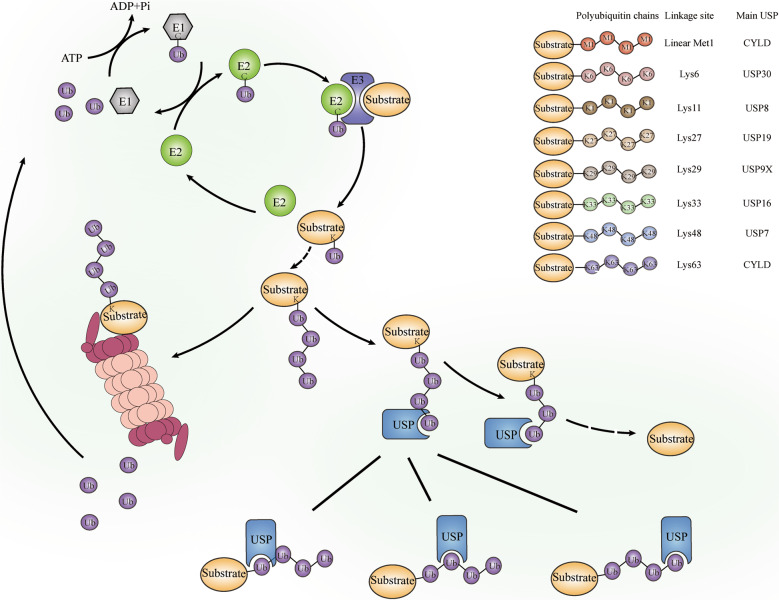
The role and mechanism of USPs family. During the ubiquitination process, the cysteine group of the ubiquitin-activating enzyme (E1) conjugates the C-terminal carboxyl group of ubiquitin to initiate ubiquitination with assumption of an ATP. Subsequently, the ubiquitin-conjugating enzyme (E2) binds to E1 and activated ubiquitin complex, catalysing the ubiquitin transfer from E1 to the active site of E2 through transesterification reaction. Then the ubiquitin-protein ligase (E3) establishes an isopeptide bond with C-terminal glycine on ubiquitin and lysine on substrate to form a ubiquitin-substrate complex. Ubiquitin can be conjugated by another ubiquitin to form a polyubiquitin chain via seven Lys residues (Lys6, 11, 27, 29, 33, 48 and 63) or Met1 by E1/E2/E3 recruitment. Once marked by a ubiquitin chain especially Lys48-linked chain, substrate tends to be degraded by proteasome. However, this process can be reversed by DUBs including multiple USPs. For example, CYLD can deubiquitinates Met1- and Lys63-linked polyubiquitin chains for signalling [29]; USP30, the only human USP that inserted in the outer mitochondrial membrane, deubiquitinates Lys6-linked di-ubiquitin for mitophagy [112]; USP8 deubiquitinates Lys11 for autophagy regulation [113]; USP19 deubiquitinates Lys27-linked polyubiquitination for innate immune responses [114]; USP9X is implicated to deubiquitinate Lys29-linked polyubiquitination [115]; USP16 deubiquitinates Lys33-linked polyubiquitination for signalling [40]; USP7 deubiquitinates Lys48-linked polyubiquitin chains for suppression of proteasomal degradation [45]. With specific USP domain, USPs can recognized the ubiquitin and catalyse the cleavage of polyubiquitin chains in the proximal, middle or distal linkage.

Ubiquitin, a small conserved regulatory protein composed of 76 amino acids, covalently attaches to substrates to act as a modular marker. During ubiquitination, substrate proteins can be attached to a monoubiquitin or to polyubiquitin chains, via multiple interaction modes. Four types of substrate ubiquitination provide additional diversity: monoubiquitination, multi-monoubiquitination, homotypic polyubiquitination, and heterotypic polyubiquitination. Ubiquitin can be linked by one of seven lysine residues (Lys6, Lys11, Lys27, Lys29, Lys33, Lys48 and Lys63), or by the first methionine (Met1) in a polyubiquitin chain [12]. As a regulatory post-translational modification, ubiquitination is reversible via DUBs, peptidases that can cleave ubiquitin from substrate proteins (Fig. 1). DUBs regulate eukaryotic ubiquitination dynamics by cleaving ubiquitin or ubiquitin-like proteins from target proteins or pro-proteins, preventing 26S proteasome degradation of the substrates [7]. As the largest subgroup of DUBs, USPs recognise a variety of substrates with Lys48-, Lys63- and Met1-linked ubiquitin chains, and regulate various biological processes, including cell migration, tumorigenesis, immunity response and inflammation activation [13]. Aberrant expressions, or blocked activity of USPs commonly cause abnormal tissue development and a series of disorders, such as abnormal expression of pro-inflammatory factors and impaired intestinal barrier function [14, 15].

### USP regulation

USPs can be controlled at the synthesis and degradation levels. USP regulation during synthesis via mRNA expression is demonstrated by the antagonistic regulation of USP22 and USP44 mRNA expression, which ensures faithful stem cell differentiation [16, 17]. Moreover, DUB transcription is upregulated in specialised cell types or at various differentiation stages, providing the potential regulation of USP levels [18]. Finally, USP activation can be modulated by post-translational mechanisms, primarily via phosphorylation. For instance, AKT activation promotes the phosphorylation, and the subsequent subcellular localisation in the membrane and cytoplasm, of USP4 [19]. PINK1-induced phosphorylation of ubiquitin at Ser65 severely alters the ubiquitin electrostatic potential and surface properties via the addition of a negative charge, consequently inhibiting the function of USP2, USP8, USP15 and USP30 [20]. In addition, substrate binding induces conformational changes, thus regulating USP activation. Binding to ubiquitin produces rearrangements in the USP catalytic triad ubiquitin-binding site, such as the realignment of the catalytic triad in USP7 [21], the translocation of two surface loops, blocking loops 1 and 2, in USP14 [22], and the movement of the finger domain in USP8 [23]. USP activation can also be regulated by scaffold or adaptor binding, such as USP14 inactivation via blockage by two surface loops until it is properly localised and associated with the proteasome [8].

## USPs and inflammation signalling pathways

The prominent role of USPs in the molecular mechanism of multiple pathways is demonstrated by their regulation of protein activation via the dissociation of monoubiquitin or polyubiquitin chains from ubiquitinated substrates. Here, we summarise relevant associations between USPs and inflammatory pathway signals, which may elucidate the regulatory role of USPs.

### USPs and the NF-κB signalling pathways

NF-κB is a family of structurally related eukaryotic protein transcription factors that comprise p50/p105, p52/p100, RelA/p65, c-Rel and RelB [24]. Under basal conditions, NF-κBs interact with NF-κB inhibitory proteins (IκBs) in the cytoplasm, forming a deactivated trimer [25]. Multiple extracellular signals activate a cytoplasmic IκB kinase complex (IKK) which phosphorylates inhibitory IκBs, leading to the separation of IκBs from NF-κB and the dissociation of IκBs via the ubiquitin-proteasome dependent pathway [26]. Stimulated NF-κBs migrate into the nucleus, bind to the DNA consensus, and activate the expression of target genes [24]. Studies have demonstrated that the NF-κB signalling pathway is essential for inducing pro-inflammatory gene expression, regulating the inflammasome, and activating inflammatory T lymphocyte and innate immune cell differentiation in the inflammatory colon of IBD patients [27, 28].

**Fig. 2:**
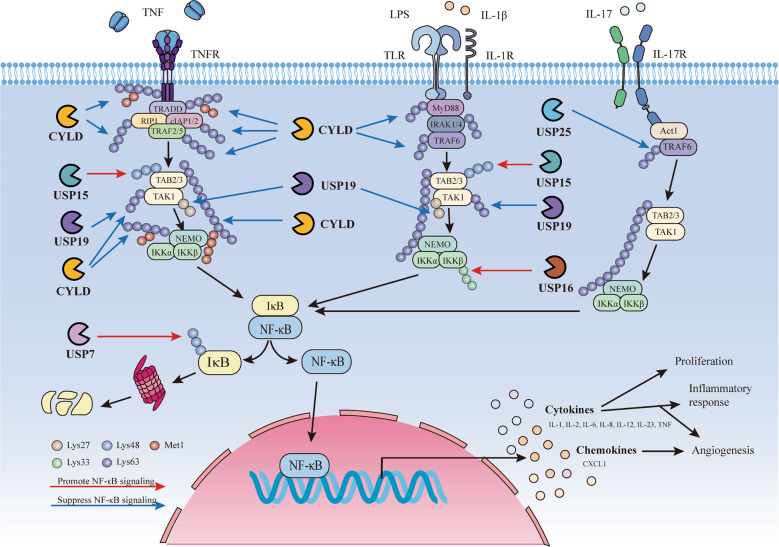
USPs and NF-κB signalling. In response to TNF, TNFR conjugates with TRADD, recruiting the kinase RIPK1 and ubiquitin E3 ligase TRAF2/5. TRAF2 associates with cIAP1 and 2 to modify multiple components in TNFR1 complex with Lys63 polyubiquitin (left side of the figure). Stimulation of Toll-like receptors (TLR) or interleukin-1 receptor (IL-1R) induces the arrangement of the Myddosome complex consisting of MyD88, IRAK4, IRAK1. Subsequently, IRAK1 is phosphorylated by IRAK4 and subsequently recruits the ubiquitin E3 ligase TRAF6 (middle side of the figure). Upon the stimulation of IL-17, IL-17R engages Act1 to mediate the recruitment of TRAF6. In all these cases, ubiquitination serves to recruit TAB2/TAB3/TAK1 and then NEMO/IKK kinase complexes, activating NF-κB signalling (right side of the figure). USPs including CYLD, USP7, USP15, USP16, USP19 and USP25 counteract the NF-κB signalling. CYLD removes Lys63-linked polyubiquitin chains from several substrates such as TNFR, TRADD, RIP1, TRAF2/5, cIAP1/2, MyD88 and TRAF6, negatively regulating NF-κB activation. USP15 deubiquitinates Lys48-linked polyubiquitination to maintain TAB2 stability and enhance NF-κB signalling induced by TNF and IL-1β. Similarly, upon TNF and IL-1β stimulation, USP19 removes Lys63- and Lys27-linked polyubiquitin chains from TAK1 and negatively regulates the activation of NF-kB. USP16 deubiquitinates Lys33-linked polyubiquitination from IKKβ and activates NF-kB. In IL-17 induced signalling, USP25 deubiquitinates Lys63-linked ubiquitination of TRAF6 mediated by Act1 and negatively regulates NF-κB signalling. Instead of regulating signals in the upstream of NF-κB, USP7 interacts with NF-κB subunits and deubiquitinates Lys48-linked ubiquitin chains, inhibiting p65 degradation and promoting NF-κB signalilng.

The USP family exerts crucial functions in regulating the NF-κB signalling pathway (Fig. 2). CYLD specifically diverts the Lys63- and Met1-linked polyubiquitin chains through two steps of cleavage reaction: initial binding and subsequent hydrolysis [29]. CYLD deubiquitinates various key negative regulators in the NF-κB pathway, including the NF-κB essential modulator (NEMO), receptor-interacting protein 1 (RIP1), TNFR-associated factor (TRAF)2, TRAF6, TRAF7, TNF receptor type 1-associated death domain protein (TRADD), RIP kinase(RIPK)1 and RIPK2 [29, 30]. Previous studies have demonstrated that CYLD causes TRAF2 ubiquitination reduction, and that a CYLD active site increases ubiquitinated TRAF2 levels, leading to TRAF2-mediated NF-κB activation, suggesting that CYLD inhibits NF-κB signalling activation through deubiquitination in TRAF2. A similar inhibitory effect has also been observed in NF-κB activation mediated by TRAF6 [31]. TNF-stimulated CYLD-deficient A549 cells exhibit increased Met1- and Lys63-linked ubiquitination of TNFR1, RIP1 and TRADD, suggesting that, when present in the TNFR signalling complex, these proteins carry Met1- and Lys63-linked ubiquitin chains, which can be antagonized by CYLD [32]. The T cells of CYLD-deficient mice overexpress p50, RelA, RelB and p100, which may be attributed to the absent inhibitory role of CYLD in TAK1 ubiquitination [33]. Upon anti-CD3 stimulation, CYLD-deficient mice further presented enhanced NEMO ubiquitination and elevated NF-κB activation, revealing the role of CYLD as a NEMO deubiquitinase and NF-κB negative regulator [34]. Containing the coil coiled 2-leucine zipper-zinc finger domain at the C-terminus, NEMO binds preferentially to Lys63-linked polyubiquitin chains, which can then be disconnected by CYLD. The Lys63-specific CYLD deubiquitinating activity removes the Lys63-linked ubiquitin chain from NEMO on RIP1, maintaining the IKKα-IKKβ-NEMO trimer structure and forming the RIP1–Fas-associated protein with the death domain (FADD)–caspase-8 complex, which participates in the negative feedback loop of NF-κB activation [35]. The loss of CYLD protects against TNF-α-induced apoptosis [36]. In addition, CYLD targets Lys63-linked ubiquitin chains on MyD88 during inflammation induced by *Haemophilus influenzae* infection [37].

In addition to CYLD, other USPs also regulate the NF-κB signalling pathway. USP15 can remove Lys48-linked ubiquitination chains and subsequently inhibit the degradation of TAB2 and TAB3 to hamper autophagy cargo receptor 1-mediated selective autophagic degradation [38]. In accordance with the results, USP15 overexpression in 293T cells enhances the interleukin (IL)-1β- and the TNF-α-mediated phosphorylation of two NF-κB activation hallmarks, the IKK complex and IκBα, while USP15 knockdown suppresses NF-κB activation and downstream gene transcription [38]. USP19, an endoplasmic reticulum–anchored DUB, is implicated in regulating endoplasmic reticulum-associated protein degradation and damage repair and is mediated by IL-1β and TNF-α at the TAK1 level, similar to USP15. USP19 negatively regulates TAK1-TAB1-dependent, rather than IKK-β- and p65-mediated, NF-κB activation with specific deconjugation of Lys63- and Lys27-linked polyubiquitin chains from TAK1 [39]. USP16 expression levels are significantly raised in macrophages of IBD patients and higher in inflammatory sections [40]. Mass spectrometry reveals that USP16 specifically binds to IKK-α and IKK-β. Under lipopolysaccharide or TNF-α stimulation, competing with NEMO, USP16 specifically deubiquitinates the Lys33-linked polyubiquitination of IKKs and promotes IKK-β-mediated p105 phosphorylation without direct IκBα or p65 phosphorylation, leading to an autoimmune response and IBD colon cancer progression [40]. USP16 levels may be enhanced by p65 overexpression on the binding sites of USP16-NF-κB2/3/4 on USP16 mRNA [41]. In addition, mRNA expression of inflammatory cytokines such as TNF, IL-1β, -12α, -12β and -23α, is elevated in the colons of USP16-deficient mice [40]. The dysregulation of nucleotide-binding oligomerization domain-containing protein 2 (NOD2), a significant CD pathogenesis genetic risk factor and a bacterial cell wall component sensor, in the colon leads to a chronic relapsing inflammatory disorder and microbial infection control failure, thereby initiating systemic immune responses and aberrant inflammation [42]. USP8 suppresses NOD2 activation stimulated by *Staphylococcus aureus* in the NF-κB pathway, thus reducing the expression of pro-inflammatory factor IL-8 [43]. USP25 is also a significant modulator in regulating NF-κB signalling. Upon IL-17 stimulation, USP25 deubiquitinates the Lys63-linked TRAF5 and TRAF6 ubiquitination mediated by Act1. Overexpression of USP25 inhibits the IL-17-mediated phosphorylation and degradation of IκBα, thereby negatively regulating NF-κB signalling [44]. USP7 knockdown inhibits p65 recruitment to the IL-6 and TNF-α promoters, suggesting USP7 regulates NF-κB at the proximal point near the promoter in place of the point upstream of Toll-like receptor (TLR)- or TNFR-mediated signalling pathways. USP7 inhibits p65 ubiquitination and Lys48-linked ubiquitin chain-mediated p65 degradation and can directly interact with NF-κB, including the p50, p52, c-Rel and RelB subunits [45]. These researches collectively represent the essential functions of USPs in affecting NF-κB signalling pathway component activation.

### USPs and the TGF-β signalling pathway

The immunosuppressive cytokine TGF-β signalling pathway has been studied with respect to potent regulatory and inflammatory activity [46]. The TGF-β ligand binds to specific TGF-β type I or II receptors on the surfaces of immune and epithelial cells to initiate TGF-β signalling. Type I receptors are recruited to type II receptors and activated via phosphorylation modification. Activated type I receptors then phosphorylate and activate the intracellular transduction signalling pathway via SMAD-dependent and -independent noncanonical pathways [47]. In the canonical SMAD-dependent pathway, TGF-β type I receptors phosphorylate SMAD2 and SMAD3, subsequently assembling SMAD4, which transfers into the nucleus to control target gene expression associated with the TGF-β signalling pathway [48]. In SMAD-independent pathways, TGF-β receptors activate other signalling modules, for instance, mitogen-activated protein kinases (MAPKs), phosphatidylinositol 3-kinase-Akt and p21 activated kinase 2 [49].

**Fig. 3:**
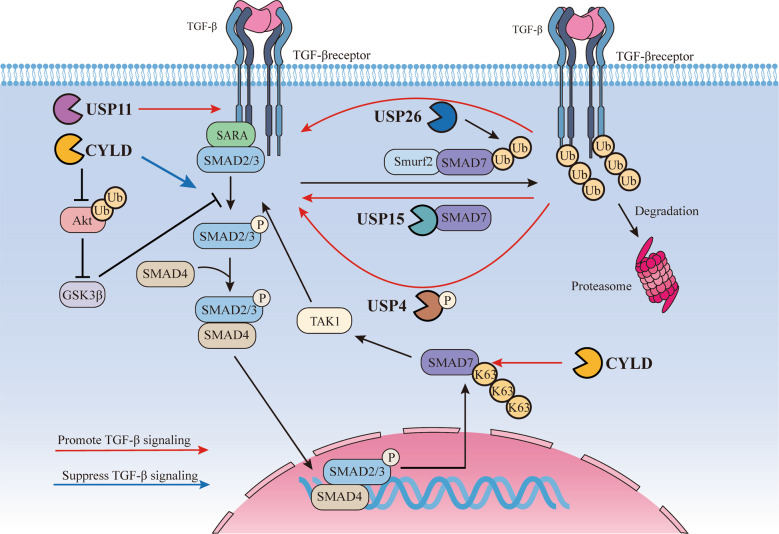
USPs and TGF-β signalling. TGF-β ligands bind to the TGF-β receptors and activate the SMADs proteins. USPs including USP4, USP11, USP15, USP26 and CYLD are associated with TGF-β signalling. Upon AKT-mediated phosphorylation, phosphorylated USP4 recruits to the activated TGF-β type I receptor directly and reverses receptor ubiquitination, leading to TGF-β signalling. USP11 deubiquitinates and stabilizes TGF-β type II receptor to promote TGF-β signalling. Reversed the polyubiquitination by SMAD specific E3 ubiquitin protein ligase 2 (Smurf2) and SMAD7, USP15 is recruited to the TGF-β type I receptor with scaffold protein SMAD7 as well and suppresses polyubiquitination and degradation of the receptor, positively regulating TGF-β signalling. USP26 is recognized as a negative regulator that deubiquitinates SMAD7 and stabilizes the interaction of SMAD7 and Smurf2, negatively regulating TGF-β signalling. CYLD decreases SMAD3 protein stability via an Akt-glycogen synthase kinase3β-hsc70-interacting protein-dependent manner, suppressing the activation of TGF-β signalling. CYLD also deubiquitinate Lys63-linked polyubiquitin chains of SMAD7 to regulate the activation of SMAD7-TAK1-TAB2/3 complex and transcription factor activator protein 1.

The negatively regulated inhibition of receptor-activated SMADs by ubiquitin ligases is counteracted by USPs through deubiquitination (Fig. 3). USP15, a TGF-β signalling pathway regulating factor in mammalian cells [50], operates both nucleus- and cytoplasm-localised downstream SMAD activation, which targets ubiquitinated receptor-activated SMADs [50]. Similar to TGF-β type I receptor associated with USP15, rather than binding to the receptor directly, USP15 binds to the receptor by SMAD7, a scaffold that also recruits SMAD-specific E3 ubiquitin protein ligase 2 (SMURF2). USP15 can remove the polyubiquitin chains of the SMURF2-ubiquitinated receptor [51]. The TGF-β signalling pathway exhibits enhanced expression of both SMAD7 and USP16; the latter, involved in negative regulation of the TGF-β signalling pathway [52], can deubiquitinate as well as stabilise the former, resulting in SMAD7-SMURF2 interaction stabilisation. USP4, with strong TGF-β signalling-inducing capability, is relocated from the nucleus to the cytoplasm and membrane by AKT-mediated phosphorylation and directly binds to, and reverses the ubiquitination of, the activated TGF-β type I receptor [19]. USP11 is a protein which participates in TGF-β signalling by regulating multiple responses, including regulatory T (Treg) and T helper (Th)17 cell induction, which is implicated in colon cancer [53]. In stable and peripherally induced Treg cells, USP11 expression is enhanced and increases TGF-β signal sensitivity in receptor-stimulated CD4+ T cells. However, increasing exogenous TGF-β or IL-2 levels do not affect USP11 expression in CD4+ T cells [53]. In addition, USP11 has been shown to affect TGF-β type II receptor stability via deubiquitination [54]. CYLD is a DUB which controls the TGF-β signalling pathway and decreases SMAD3 protein stability in an Akt-glycogen synthase kinase3β-hsc70-interacting protein-dependent manner, thus inhibiting TGF-β signalling [55]. CYLD-deficient T cells exhibit an elevated TGF-β responsiveness capacity and promote AP-1, TAK1 and p38 activation. Furthermore, CYLD deubiquitinates SMAD7 Lys63-linked polyubiquitination chains, regulating activation of the SMAD7-TAK1-TAB2/3 complex and transcription factor activator protein 1 [56].

### USPs and other signalling pathways

In addition to the NF-κB and TGF-β pathways, the USP deubiquitinating role is essential to other signalling pathways. USP11 promotes the MAPK signalling pathway in a PPP1CA-dependent manner [57]. PPP1CA, one of the three catalytic subunits of protein phosphatase 1, is implicated in tumorigenesis via the MAPK signalling pathway [58]. A previous research demonstrated that elevated USP11 in colorectal cancer cells could protect PPP1CA from proteasome-mediated degradation, thus activating the MAPK pathway and promoting colon cancer cell growth and metastasis [57]. Moreover, per the critical protein ubiquitination function in TLR signalling, several USPs regulate TLR-mediated signalling via crosstalk with other pathways. USP25 inhibits TLR4-dependent activation of NF-κB and MAPKs to regulate innate immune responses by deubiquitinating adaptor protein TRAF3 via the coiled-coil domains, along residues 644 to 1055 [59]. USP15 interacts with Hrd1, an E3 ligase, in microphages to regulate IkBα degradation and pro-inflammatory cytokine production in the TLR4-mediated signalling pathway [60]. In small intestine tumour tissues or USP25-deficient colons, the level of Wnt-related gene products is significantly reduced compared to those in USP25-sufficient colons subjected to Wnt3a stimulation, demonstrating that USP25 participates in Wnt signalling pathway regulation during colonic tumourigenesis [61]. Finally, USP28 controls cellular activation levels of the Notch pathway, which maintains intestinal homoeostasis and controls intestinal stem cell development and differentiation [62]. Notch signalling regulator alterations can lead to aberrant Notch activation and subsequent severe complications, including intestinal inflammation due to secretory cell deficiency [63]. USP28 recognises and deubiquitylates Notch receptor 1, regulating the gene expression of Notch signalling pathway downstream molecules, such as Hes1, Hes5 and delta-like canonical notch ligand 1 [64]. Insufficient USP28 levels may deactivate excessive intestinal hyperplasia and impaired intestinal differentiation [65].

USPs are also involved in regulating the Hippo pathway, a conserved kinase cascade that has been implicated in colon development, intestinal regeneration and homoeostasis [66]. Yes-associated protein (YAP)1 and the transcriptional co-activator with PDZ-binding motif, both downstream effectors of the Hippo pathway, are the key transcriptional co-activators that regulate target gene expression and tissue growth. YAP is elevated in IBD and is mainly found in intestinal epithelial cell (IEC) nuclei in the villi and crypts of the small intestine and colon [67, 68]. USP9X strongly interacts with large tumour suppressor kinase 1, an important YAP negative regulator, consequently stimulating the Hippo pathway [69]. In addition, USP47 interacts with YAP, promotes YAP transcriptional activity and protein stability, and is the interactor for β-Trcp, an E3 ubiquitin ligase, leading to NLR family pyrin domain-containing (NLRP)3 proteasomal degradation via Lys27-linked polyubiquitination at Lys380. YAP binds to, and promotes activation of, NLRP3, which competitively inhibits β-TrCP1-mediated polyubiquitination [70], demonstrating YAP’s function in immune disorders and inflammatory diseases. This suggests that the USP47-YAP signalling axis in the Hippo pathway may mediate NLRP3 activation and regulate immune and inflammatory responses.

## USPs and IBD pathogenesis

Although the causes of IBD pathogenesis remains elusive, many studies have revealed that genetic susceptibility, intestinal epithelial barrier, mucosal immune response, and gut microbiota are associated with an altered homoeostasis, leading to the aggravation of gastrointestinal inflammation. In this chapter, we summarise the role of USPs in the pathogenesis of IBD (Table 1).

**Table 1:** USPs and pathogenesis of IBD.

USPs	Pathogenesis of IBD	Expression	Mechanism/major finding	Patients/Model	References
USP7	Imbalance of intestinal immunity	Deficiency	Decreased ability of resolving inflammation	Adoptive-transfer-induced colitis	[102]
USP8	Imbalance of intestinal immunity	Deficiency	Disturbed T cell homoeostasis, impaired T cell regulatory function, predominance of CD8+ γδ T cells	T cell-specific USP8-deficient colitis in mice	[103]
USP9X	Defect of intestinal barrier	Decrease	Decreased FBW7, tissue damage	DSS-induced colitis	[91]
USP9X	Defect of intestinal barrier	Inactive	Impaired intestinal regeneration	Colitis-associated intestinal cancer	[91]
Usp22	Defect of intestinal barrier	Deficiency	Moderate and severe epithelial damage; increases local and systemic inflammation	DSS-induced colitis	[90]
Usp22	Imbalance of intestinal immunity	Deficiency	Increases IL-6 levels; increases local immune cell infiltration and systemic inflammation in CD45-positive immune regulatory cells	DSS-induced colitis; inflammation-associated CRC murine model	[90]
USP25	Defect of intestinal barrier	Deficiency	Increased Paneth cells and IECs, epithelial damage	DSS-induced colitis	[61]
USP25	Imbalance of intestinal immunity	Deficiency	Higher expression of IRF-dependent genes and genes related to inflammatory cytokines and chemokines	DSS-induced colitis	[61]
USP25	Imbalance of intestinal immunity	Deficiency	increased phosphorylated p38 and p65, decreased TRAF3 and elevated IL-6 and TNF-α	Colitis infected by Citrobacter rodentium	[61]
CYLD	Defect of intestinal barrier	Deficiency	Histologic damage, greater leucocyte infiltration, histologic damage, and increased intestinal epithelial dysplasia	AOM and DSS-induced colitis-associated cancer model	[34]
CYLD	Defect of intestinal barrier	Deficiency	Enhanced bacterial dissemination, greater submucosal oedema and broader mucosal impairment and ulceration	Citrobacter rodentium induced colitis	[88]
CYLD	Imbalance of intestinal immunity	Deficiency	Higher concentration of IL-18	Colitis infected by Citrobacter rodentium	[88]
CYLD	Imbalance of intestinal immunity	High/low gene expression	Low/high production of IL-18	Patients with UC	[88]
CYLD	Defect of intestinal barrier	Inactive	Decreased intestinal epithelial cell death	Colitis in mice by FADD deficiency in IECs	[89]
CYLD	Imbalance of intestinal immunity	Deficiency	Increased cytokines including IL-10	Colitis with transferred T cell	[33]
sCYLD	Imbalance of intestinal immunity	Increase	Enhanced SMAD7 translocation, impaired suppressive function of Treg cells	sCYLD/SMAD7 mice	[98]
CYLD	Disturbance of gut microbiota	Decrease	Increased invasion and intracellular replication of AIEC bacteria.	Transfected T84 IECs	[77]
CYLD	Disturbance of gut microbiota	Increase	Decreased number of AIEC bacteria	Transfected T84 IECs	[77]
USP1	Genetic susceptibility	–	SNP (rs1748195) in USP1 gene is associated with CD risk	Patients with CD	[83]
USP3	Genetic susceptibility	–	Polymorphisms in USP3 genes are associated with both CD and UC	Patients with IBD	[77]
USP4,	Genetic susceptibility	–	Polymorphisms in USP4 genes are associated with both CD and UC	Patients with IBD	[74, 77, 82]
USP3, USP5, USP15, USP19, USP39	Genetic susceptibility	–	Polymorphisms in USP5, 15, 18, 39 genes are associated with UC	Patients with UC	[82]
USP25	Genetic susceptibility	–	Two SNPs, rs7278277 and rs2242830, are associated with CD and IBD, respectively	Patients with IBD	[79]
USP40	Genetic susceptibility	–	Polymorphisms in USP4 genes are associated with both CD and UC	Patients with IBD	[74, 77, 82]
USP44	Genetic susceptibility	–	USP44 methylation relevant to neoplasia associated IBD	Patients with neoplasia associated IBD	[81]
CYLD	Genetic susceptibility	–	The most important gene of ubiquitin proteasome system that associated with CD	Patients with CD	[76–78]

### USPs and genetic susceptibility

Since 2001, numerous studies on the genetic component of IBD susceptibility have identified over 240 IBD-related loci [71]. Genetic susceptibility data has helped shape the emerging understanding of IBD as a system-level dysfunction in both mucosal immune and commensal ecosystems and thus, identify therapeutic intervention targets [3]. Studies have shown that ubiquitin- and deubiquitin-related genes have strong associations with IBD, for instance, a coding variant *333F in *Clorf106* increases IBD risk [72]; a single-exon finger E3 ubiquitin-protein ligase, *RNF186* is associated with UC susceptibility [72], and the protein-truncating R179X variant in *RNF186* exerts a protective effect against UC [73]. Furthermore, polymorphisms in the human DUB gene *A20*/*TNFAIP3* locus are associated with IBD and negatively regulate inflammatory cytokines [74]; associations of *CARD9* rs10870077 SNP to CD an UC are also observed [75]. Except these gene associations, USP gene variants that have been identified as related to IBD susceptibility include variants of *CYLD* [76, 77]. In a multicentre cohort study, *CYLD* was demonstrated to be the most significant CD-related ubiquitin-proteasome system gene. The single nucleotide *CYLD* polymorphism rs12324931 demonstrates the strongest association with CD. Genetic stratification research also indicates that *CYLD* likely plays a vital role in CD patients [78].

Meta-analysis has detected an IBD-relevant single nucleotide polymorphism near *USP25*, which is located on chromosome 21q11 [79] and, according to genome-wide association studies (GWASs), displays genome-wide significant associations for IBD patients of various ethnicities, including African American, Caucasian, and Korean [74, 79, 80]. An ImmunoChip analysis demonstrated USP25 is related to CD in Korean populations [80]. In a methylation-specific curve biopsy analysis, USP44, encoded by a gene on chromosome 12, was identified as a possible DNA methylation signature contributing to the early detection of colorectal cancer associated with IBD. However, the correlation between USP44 methylation and IBD-associated colorectal cancer was only detected in IBD patients with associated neoplasia, and not in patients with sporadic colorectal cancer [81]. GWASs by Jostins et al. and Cleynan et al. have revealed a USP4 association with both UC and CD and a USP40 association with CD only [74, 82]; the latter study also identified five USPs: USP19, USP15, USP39, USP3 and USP5, associated with UC only [82]. However, a later study by Cleynan et al. reached a slightly different conclusion that USP3 and USP40 are associated with both UC and CD, but USP4 is only related to UC [77]. A trans-ethnic association study has revealed that USP1 single nucleotide polymorphisms are associated with CD in both European and non-European populations [83].

### USPs and the intestinal epithelial barrier

The intestinal epithelial barrier is composed of multiple physical, cellular, and chemical components, primarily contributed by intact IEC coherence—robust intercellular junctions including tight junctions, adherens junctions, and desmosome—and by mucus secreted by goblets [84, 85]. Acting as the guardian of the mucosal surface, the intestinal epithelial barrier not only regulates the bidirectional flow of water, macromolecules, and ions between the lumen and the host, but also protects intestinal cells from antigens and microbes in the lumen [85]. Intestinal barrier dysregulation will contribute to IBD pathology. Both CD and UC have common features, including epithelial breaks, reduced tight junction expression, and glandular atrophy [86]. Impairment of the intestinal barrier function generates increased permeability, resulting in an aberrant interaction between luminal pathogens and the intestinal mucosal immune system [87]. In genetically susceptible individuals, impaired gut barrier dysregulates immune responses and perpetuates chronic autoinflammation, contributing to IBD [84].

Studies have revealed that CYLD-deficient mice are susceptible to colitis. Upon azoxymethane and DSS administration, CYLD-deficient mice demonstrate increased colonic epithelium dysplastic changes, greater leucocyte infiltration, and histologic damage [34]. In CYLD-deficient mice with *Citrobacter rodentium-*induced severe inflammation, intestinal barrier disruption is demonstrated by enhanced bacterial dissemination, greater submucosal oedema, broader mucosal impairment and ulceration [88]. Endoscopic and histological analyses of mice with an IEC-specific deficiency of FADD, an adaptor protein involved in apoptosis initiation, have revealed severe colitis with epithelial erosion, mucosal thickening and transmural inflammation that may be due to CYLD catalytic activity. CYLD inhibition protects epithelial cells from death and prevents commensal bacterial infection and intestinal epithelial barrier disruptions that trigger colitis development [89]. The abnormal response of mice CYLD-deficient T cells spontaneously develops colitis and displays an inflammatory phenotype characterised by mucosa thickening, inflammatory cell infiltration, and goblet cell depletion, and also exhibits substantial weight loss and high pro-inflammatory cytokine levels in the colon [33]. In an azoxymethane and DSS-induced colitis-associated cancer model, CYLD-deficient mice are susceptible to colonic inflammation with histological damage, greater leucocyte infiltration, increased dysplastic changes and more mucosal ulcers in the intestinal epithelium [34].

Other USPs also function in the intestinal epithelial barrier in IBD cases. Kosinsky et al. demonstrated that USP22 maintains the intestinal epithelial integrity in DSS-induced colitis. In this mouse model, the USP22-deficient group presented more severe epithelial damage [90]. USP9X-deficient mice with DSS-induced colitis exhibit decreased body weight, indicating impaired gut regeneration. In a colitis-associated intestinal cancer model, USP9X inactivation impairs intestinal regeneration. In DSS-induced colitis, the USP9x and FBW7 protein levels are obviously decreased in the peak phase of colitis and return to normal levels during later phases. Notably, decreased FBW7 protein levels are a consequence of reduced USP9x expression. In accordance with these findings, USP9x suppresses c-MYC via FBW7 transcriptional activity, controlling intestinal tissue homoeostasis during tissue maintenance, damage, and repair [91]. USP25, expressed ubiquitously in various intestinal cell types, plays an essential role in intestinal inflammation [92, 93]. When subjected to DSS-induced colitis, the intestines of USP25-deficient mice exhibit increased quantities of Paneth and goblet cells in the villi, and greater damage to the remaining epithelium and intestinal epithelium than those of USP25-sufficient mice [61]. TLRs overexpress in goblet cells, Paneth cells and IECs [94]. Therefore, increased secretory cell levels and elevated TLR signalling in secretory cells and IECs may contribute to the protection against DSS-induced colitis in USP25-deficient intestines [61].

### USPs and intestinal immunity

The intestinal immune system consists of innate and adaptive immune responses. Innate immune cells, equipped with germline-encoded receptors which recognise conserved pathogen-associated molecular patterns, include macrophages, granulocytes and dendritic cells. Adaptive immune cells include adaptive B and T lymphocytes, which contain antigen-specific receptors with tailored responses to multitudinous antigens, and mucosal-associated invariant T cells, which demonstrate a limited diversity of antigen receptors [2, 95]. The immune system maintains an intestinal homoeostasis balance between tolerance toward commensal microflora and aggressive responses to invading pathogens, which relies on mucosal immune cells, commensal bacteria, and IECs. Under physiological conditions, macrophages and Treg cells secrete TGF-β and IL-10 to induce immunotolerance [96]. However, under pathological conditions, complex networks of elevated pro-inflammatory cytokines, such as IL-1, -4, -6, -12, and -23, enhancement of Th1, Th2 and Th17 cells, and activation of other immune cells, cooperatively trigger IBD [96].

Studies have shown that CYLD levels in IBD patient mucosae are lower than those in normal samples [77, 97]. CYLD deubiquitinates the NLRP6 Lys63-linked polyubiquitin chains and inhibits the NLRP6-apoptosis-associated speck-like protein comprising a CARD inflammasome complex, which prevents active IL-18 concentration in colonic epithelial cells [88]. Following *Citrobacter rodentium* infection, CYLD-deficient mice exhibit a substantially higher IL-18 concentration, indicating that severe mice colitis depends on elevated IL-18 production. In accordance with these findings, in UC patients, CYLD mRNA expression is inversely correlated with IL-18 protein levels [88]. Moreover, CYLD catalytic activity inhibition may prevent chronic inflammation and maintain physiological immune homoeostasis [89]. CYLD-deficient IBD mouse models exhibit IBD-like phenotypes with inflammatory CD4+ T cell infiltration in inflamed areas lacking colonic patches, crypt damage and muscularis layer thickening. In transferred T cell models, the loss of CYLD induces autoimmunity and colitis. The in vitro hyper-response to T cell receptor stimulation in CYLD-deficient T cells results in hyperproliferation and markedly greater aberrant production of cytokines, including IFN-γ, indicating CYLD’s pivotal role in impeding activation of peripheral T cells. CYLD-deficient mice also express elevated levels of T cell-derived cytokines, for example, IL-4, -10 and -12, in the colon [33]. Increased CYLD splicing and SMAD7 expression are discovered in the intestinal T cells of CD patients. CYLD splicing impairs Treg and Th17 cell differentiation, increases Th1 cell polarisation, enhances SMAD7 translocation to the nucleus with cleavage of Lys63-linked polyubiquitin chains, and inhibits TGF-Β signalling. Co-overexpression of CYLD splicing and SMAD7 in T cells impairs Treg cell suppressive functions, resulting in abnormal T cell homoeostasis and triggering fatal colitis. However, these effects have not been examined in CYLD-knockout SMAD7 mice [98].

In IBD, the NLRP3 inflammasome is upregulated in the colonic mucosa [99]. The NLRP3 inflammasome serves as a central node in intestinal microbiota and inflammatory response interactions, and deubiquitination is an essential step in NLRP3 inflammasome activation, which leads to IBD pathogenesis [100]. USP7 and USP47 act as NLRP3 positive regulators, interfering with Lys63 polyubiquitin modification and regulating NLRP3 activation in microphages at a post-transcriptional level [101]. USP7 inhibition by its specific inhibitor P22077 prevents NLRP3-containing oligomer formation and decreases the release of ATP-dependent IL-1β, IL-18 and caspase-1 [101]. Highly regulated and active in Treg cells, USP7 reduces Foxp3 polyubiquitination and conversely enhances Foxp3 protein expression. In an adoptive-transfer-induced colitis model, Treg cell USP7 loss abrogates the capacity to resolve inflammation, suggesting the role of USP7 in adaptive immunity [102]. Mice with colitis developed by T cell-specific USP8 deficiency exhibit disturbed T cell homoeostasis, impaired regulatory T cell function, and predominance of CD8+ γδ T cells, revealing the critical role of T cell development and homoeostasis [103]. USP22-deficient mice in a DSS-induced colitis model display increased local immune cell infiltration and higher serum IL-6 levels. Inflammation-associated colorectal cancer murine models exhibit increased local immune cell infiltration and systemic inflammation in CD45-positive immune regulatory cells [90]. In DSS-induced colitis models, USP25-deficient mice exhibit increased leucocytes, CD8+ T cells, and CD11b^+^F4/80^+^ macrophages, and decreased infiltration of CD4^+^IL-17A^+^ T cells and CD11b^+^Gr-1^+^ neutrophil in the colonic lamina propria. USP25 loss and DSS-induced colitis resistance in nonhematopoietic cells are prevalent in immune cell homoeostasis of the colon lamina propria. This study further demonstrates that USP25 deficiency positively alters cytokine expression and protects mice against DSS-induced colitis. In addition, *Citrobacter rodentium*-infected USP25-deficient mice present raised concentration of phosphorylated p38 and p65 and of IL-6 and TNF-α, and decreased concentration of TRAF3 in colon tissues and sera [61].

### USPs and gut microbiota

The focus in the past decade on the multiplicity and complexity of gut microbiota has provided growing insights into the pathogenic and commensal microorganisms that establish and disrupt intestinal homoeostasis [104, 105]. The indigenous intestinal microbiota is considered be a major trigger of IBD [106]. Up to five major bacteria phyla, Firmicutes, Bacteroidetes, Actinobacteria, Proteobacteria and Verrucomicrobia, contain thousands of anaerobic species that colonize the intestine with a gradient driven by bile acid, oxygen and antimicrobial peptides from the proximal to the distal [107]. A systematic prospective study has revealed that IBD patients are more likely to have intramucosal bacteria in the colon and terminal ileum than control subjects [108]. Multiple studies have revealed global changes in the gut microbiota composition of IBD patients, including decreased levels of members of the Firmicutes phylum, specifically *Faecailbacterium prausnitzii* in CD patients, and increased levels of members of the Proteobacteria phylum, including *Escherichia coli* in IBD patients, suggesting an essential role of dysbiosis in disease state [109]. Recently, studies of glycosylation in the intestinal interactions of gut microbiota suggest the pivotal role of post-translational modification in IBD [110]. As one such complex post-translational modification, deubiquitination by USPs may also function in dysbiosis-mediated IBD. CD-associated adherent-invasive *E. coli* LF82 bacteria modulate CYLD protein levels, which subsequently decreases CYLD expression in transferred T84 cells, leading to increased adherent-invasive *E. coli* invasion and intracellular replication. Conversely, CYLD overexpression in T84 cells reduces adherent-invasive *E. coli* bacteria levels 24 h after infection [77]. CYLD can also dissociate Lys63-linked polyubiquitin chains from NLRP6 and hampers its activation [88]. Colitogenic microbiota with altered bacterial populations are present in the intestine of NLRP6-deficient mice, replacing intestinal microbiota in immunocompetent mice [111]. This emphasizes the role of CYLD in gut microbiota regulation and intestinal homoeostasis via NLRP6 deubiquitination.

## Conclusion

Here, we have summarised the regulatory roles of USPs in several signalling pathways and the relationship between USPs and IBD pathogenesis. Certain USPs are key IBD regulators in pro-inflammatory signalling pathways and play important roles in regulating protein activation during post-translational modification. The majority of recent IBD studies has chiefly focused on the proliferation of cellular substances, including pro-inflammatory cytokine production, epithelial-mesenchymal transformation, and aberrant fibrotic growth. However, the role of protein degradation has remained unaddressed, especially the DUB inverse process that counteracts regulatory protein proteolysis. After modification of the ubiquitination process by E1/E2/E3, ubiquitinated proteins either undergo degradation or deubiquitination by DUBs. As the largest DUB subfamily, the role of USPs should not be overlooked. Two aspects of the regulatory role of USPs are associated with the proteolytic process: impeding the normal hydrolyzation affecting the cellular metabolic cycle or blocking the natural apoptosis of the regulatory protein. We have summarised findings regarding the pivotal role of USPs in susceptibility genetics, intestinal epithelial barrier function, immunity, and gut microbiota associated with IBD. However, our current knowledge is far from adequate. The exact signals that trigger chronic inflammation are yet to be explored. Elucidating the USP control of protein modification in the regulation of protein activation and degradation provides a novel approach in identifying effective inflammatory response targets and the treatment of inflammation. Thus, targeting USPs to clarify the underlying molecular mechanism may be an effective strategy to treat IBD, and more extensive research on USPs is needed to explore their specific mechanisms.

## Data Availability

All data included in this review are available upon request by contact with the corresponding author.
